# Efficient Knockin Mouse Generation by ssDNA Oligonucleotides and Zinc-Finger Nuclease Assisted Homologous Recombination in Zygotes

**DOI:** 10.1371/journal.pone.0077696

**Published:** 2013-10-22

**Authors:** Bin Shen, Xin Zhang, Yinan Du, Jianying Wang, Jun Gong, Xiaodong Zhang, Peri H. Tate, Hongliang Li, Xingxu Huang, Wensheng Zhang

**Affiliations:** 1 MOE Key Laboratory of Model Animal for Disease Study, Model Animal Research Center of Nanjing University, National Resource Center for Mutant Mice, Nanjing, China; 2 College of Life Sciences, Wuhan University, Wuhan, China; 3 Wellcome Trust Sanger Institute, Wellcome Trust Genome Campus, Hinxton, Cambridge, United Kingdom; University of Texas Health Science Center at San Antonio, United States of America

## Abstract

The generation of specific mutant animal models is critical for functional analysis of human genes. The conventional gene targeting approach in embryonic stem cells (ESCs) by homologous recombination is however laborious, slow, expensive, and limited to species with functional ESCs. It is therefore a long-sought goal to develop an efficient and simple alternative gene targeting strategy. Here we demonstrate that, by combining an efficient ZFN pair and ssODN, a restriction site and a loxP site were successfully introduced into a specific genomic locus. A targeting efficiency up to 22.22% was achieved by coinciding the insertion site and the ZFN cleavage site isogenic and keeping the length of the homology arms equal and isogenic to the endogenous target locus. Furthermore, we determine that ZFN and ssODN-assisted HR is ssODN homology arm length dependent. We further show that mutant alleles generated by ZFN and ssODN-assisted HR can be transmitted through the germline successfully. This study establishes an efficient gene targeting strategy by ZFN and ssODN-assisted HR in mouse zygotes, and provides a potential avenue for genome engineering in animal species without functional ES cell lines.

## Introduction

Generating mouse models with specific gene mutations can be achieved by conventional gene targeting in embryonic stem cells (ESCs) by homologous recombination. This approach has been proven robust for functional analysis of human gene homologues. However, generation of mutant mice by this approach is often laborious, slow, and expensive [1]. Moreover, this approach is only applicable in animal species with established functional ESC lines (ie. Pluripotent cells which can populate the embryonic germline). To circumvent these limitations, alternative nuclease-mediated approaches such as zinc-finger nucleases (ZFNs) [2-5], transcription activator-like effector nucleases (TALENs) [6-10], or clustered regularly interspaced short palindromic repeats/CRISPR-associated systems (CRISPR/Cas9) [11,12] have been developed to generate targeted mutations in one-cell embryos by inducing double-strand breaks (DSBs) and the error-prone nonhomologous end-joining (NHEJ) DNA repair pathway. It provides an easier, quicker, and more affordable option. Nevertheless, the mutations by ZFNs, TALENs, or CRISPR/Cas9 are unpredictable owing to the variable DNA repair by NHEJ and are only useful in gene knockout studies [2-12]. 

Based on the observation that DSBs increase the rate of homologous recombination (HR) [13], conventional plasmid-based targeting vectors have been combined with ZFN [14-18], TALEN [19,20], or CRISPR/Cas9 [21] expression to achieve efficient genome editing in different cell types. Targeting vectors introduced simultaneously with ZFN, TALEN, or CRISPR/Cas9 reagents in the zygote can provide a template for high-fidelity HR and expand the versatility of nuclease-mediated genome editing. As expected, precise genome editing by ZFN- or TALEN-assisted HR in zygotes has been successfully achieved in mouse, rat, and rabbit models [5,22-25]. 

However, all previous reported attempts of gene targeting by nuclease-assisted HR in zygotes occurred at low efficiency, limiting the application of this strategy. Using single-stranded oligodeoxynucleotides (ssODNs), Chen et al. demonstrated successful genome targeting in cultured cell lines at a frequency of 1~30% without antibiotic selection [26], providing a potential strategy to improve nuclease-assisted HR in zygotes. Moreover, the design and synthesis of ssODNs is easy and intuitive as compared to the laborious construction of conventional plasmid-based targeting vectors, which often requires long regions of homology to the target locus. Bedell et al. successfully generated the first knockin zebrafish by one-cell embryo co-injection of TALENs and ssODNs [27], supporting the feasibility of genome editing by ZFN-assisted HR using ssODNs as donor in mouse zygotes. 

To test ZFN and ssODN-assisted HR in mouse zygotes, we designed and constructed an efficient ZFN pair targeting the mouse *c-kit* locus and optimized the concentration and the homology length of the donor ssODNs. We also used donor ssODNs isogenic to the targeted endogenous locus. With these optimizations, precise genome editing with efficiency as high as 22.22% was achieved. Our study establishes a quick, simplified, and efficient strategy for genome editing mediated by ZFN and ssODN-assisted HR in mouse zygotes. 

## Results

### Evaluation of ZFNc-kit in vitro and in vivo

**Figure 1:**
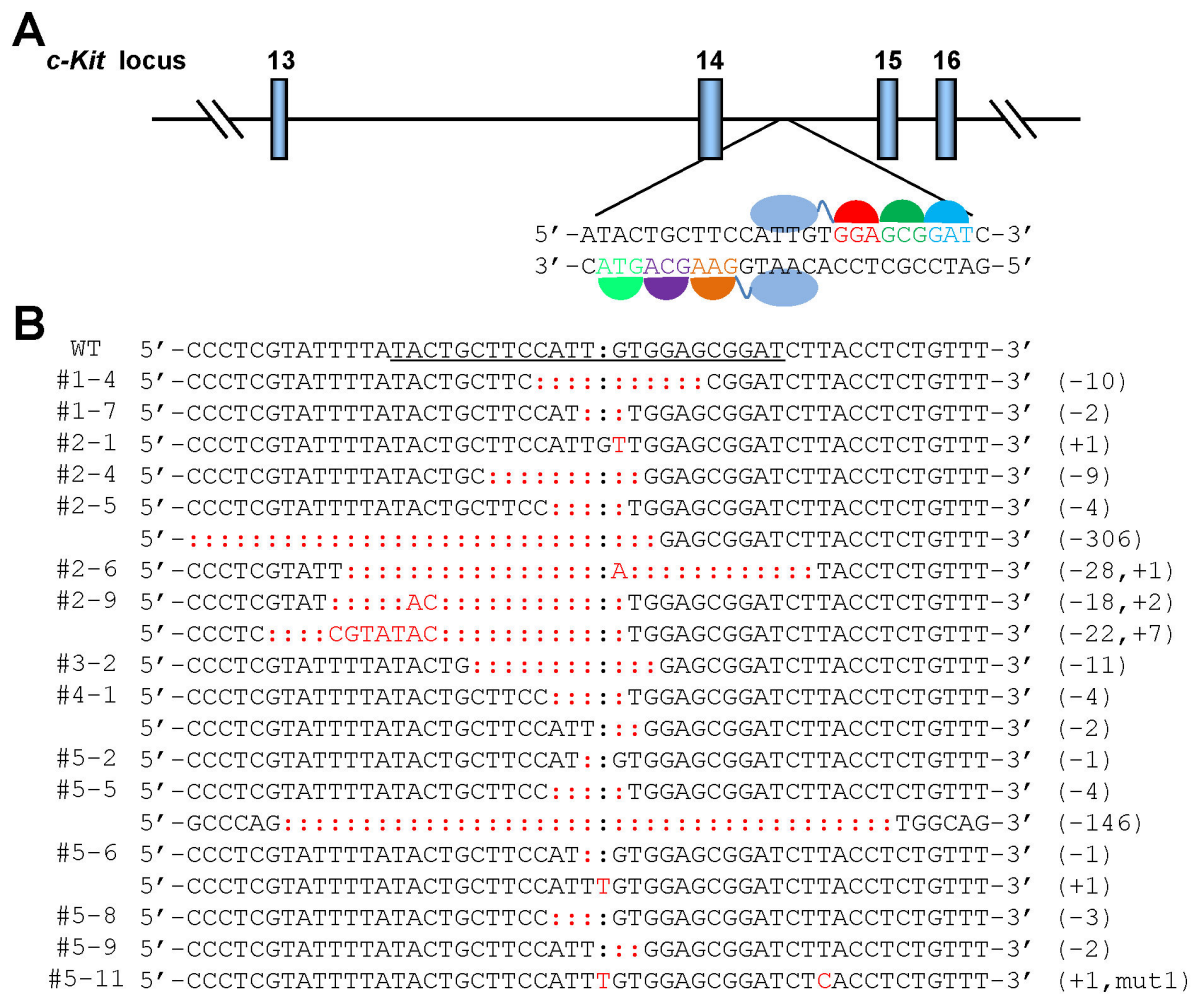
Generation of ZFN-mediated *c-kit* mutant mice. a. Schematic showing the layout of ZFN target sites. The left and right ZFN binding sites with the spacer region are shown. b. Sequence analysis of the modified *c-kit* allele in 44 founder animals. The targeted region on the mouse *c-kit* locus was PCR-amplified from genomic DNA extracted from tails of the 5-day-old F0 pups, then subjected to T-A cloning. Colonies were randomly picked for DNA sequencing. Indels and mutations were detected in 8 out of 27 founders treated with 2.5 ng/μl ZFN mRNA and 7 out of 17 founders treated with 5 ng/μl ZFN mRNA. The ZFN binding site is underlined. Indels and mutations are highlighted in red.

We first constructed a ZFN pair capable of inducing DSBs efficiently. We designed a ZFN pair (ZFNc-kit) that recognizes 18 nucleotides in the intron 14 of the mouse *c-kit* locus on chromosome 5 (Figure 1A). The targeted region in the *c-kit* locus was amplified from mouse genomic DNA and subjected to T-A cloning (T-Ac-kit). Left and right arms of the ZFNc-kit (Sequences S1) were codon optimized, synthesized, and cloned into expression vectors, pST1374 (Addgene No. 13426) and pcDNA3.1 (-) (Life Technologies), respectively. Linearized ZFNc-kit arms were transcribed to RNAs *in vitro* by T7 RNA polymerase [12]. ZFNc-kit mRNAs were modified further by capping the 5’ end and adding a poly-A tail at the 3’ end to increase resemblance to authentic mRNA. We then verified the nuclease activity of ZFNc-ckit mRNA by assessing its ability to induce indels (insertions/deletions) in naked plasmid DNA in one-cell zebrafish embryos. The purified ZFNc-kit mRNAs and the T-Ac-kit plasmid were co-injected into one-cell zebrafish embryos. The embryos were then cultured for 12 hours and harvested for total DNA extraction. 

The ZFN-targeting region on the T-Ac-kit plasmid was then PCR-amplified from the extracted DNA, subjected to T-A cloning and sequenced. Indels were detected in 27 out of 80 T-A colonies, indicating a targeting efficiency up to 33.75% for the ZFNc-kit pair. As expected, point mutations, deletions (1-37 bp) and insertions (1-25 bp) of varying sizes were observed at the target site (Figure S1B). These results confirm that our ZFNc-kit design is highly efficient in targeting the mouse *c-kit* locus. 

We then injected the ZFNc-kit mRNAs (2.5 or 5 ng/μl) into one-cell mouse embryos as performed in routine transgenic mouse generation. In total, 372 out of 431 injected embryos obtained from crossing of CBA male mice and superovulated C57BL/6J female mice were transferred into 11 pseudopregnant females (Table 1). Forty-four pups born from the injected embryos were genotyped by sequencing the target site using PCR-amplified genomic DNA extracted from tails of the 5-day-old founder animals. Double peaks appeared in samples from 15 out of 44 pups (data not shown). PCR products from the 15 pups with double peaks were then cloned and sequenced (Figure 1B). Indels and mutations were detected in 8 out of 27 (29.63%) and 7 out of 17 (41.18%) founders from 2.5 and 5.0 ng/μl ZFN mRNA microinjection respectively. These results demonstrate that we have successfully generated a functional and efficient ZFN pair targeting the mouse *c-kit* locus. 

**Table 1:** Summary of embryo microinjection of ZFN mRNA.

ZFN mRNA	Embryos injected	Embryos transferred	Recipient	Pups	NHEJ positive (mutation rate in %)	No. pups
2.5 ng/μl each	276	237	7	27	8 (29.63%)	#1-1 to #1-14, #2-1 to #2-9, #3-1 to #3-4
5 ng/μl each	155	135	4	17	7 (41.18%)	#4-1 to #4-5, #5-1 to #5-12

### Targeted Insertion of a Restriction Site into the c-kit Locus by ZFNc-kit and Gene-Targeting ssODNs

**Figure 2:**
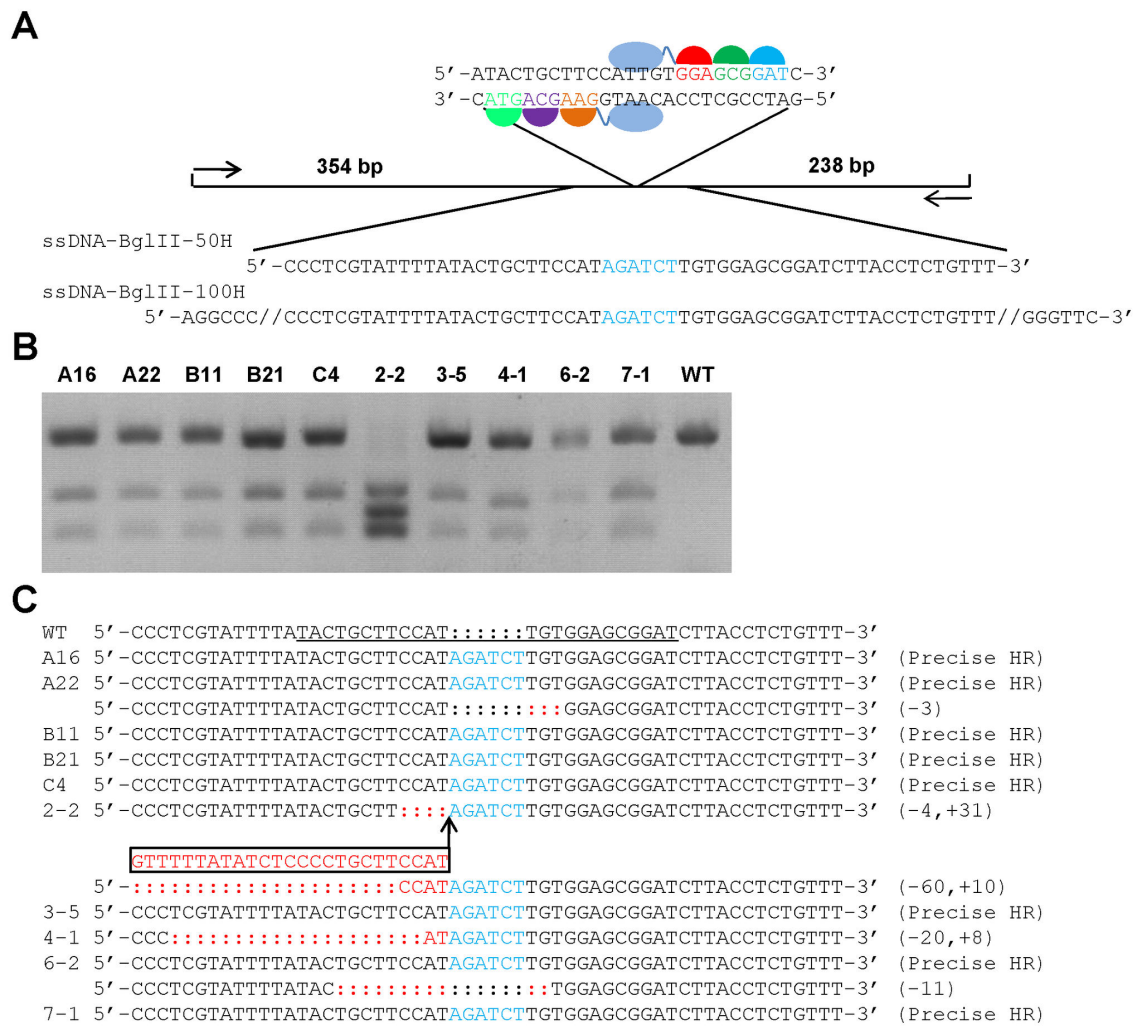
Targeted insertion of a restriction site using ZFNc-kit and gene-targeting ssODNs. a. A schematic of the mouse *c-kit* locus and the ssODN sequences of different sizes designed to introduce an exogenous BglII restriction site (in blue) into the genome *in* *vivo* using ZFNc-kit and ssODNs. b. Detection of introduced BglII site in founder animals. The representative gel electropherogram of genotyping results by BglII digestion of PCR product. F0 pups were genotyped by BglII digestion of the 592 bp PCR product amplified from tail genomic DNA. The PCR product from the wild type *c-kit* allele cannot be digested by BglII (WT), while the PCR product from the targeted mutant allele (10 out of 109 founders) can be digested by BglII into two distinct fragments (238 bp and 354 bp). The PCR primers are listed in Table S1. c. Sequence analysis of the *c-kit* mutant allele of the 10 founders with correct BglII restriction bands. The target region of the mouse *c-kit* gene was PCR-amplified from tail genomic DNA of the 5-day-old founders and subjected to T-A cloning. Colonies were randomly picked for DNA sequencing. Precise BglII site insertion was detected in 8 out of 10 mutant founders. ZFN binding site is underlined. BglII sites are highlighted in blue. Indels are highlighted in red.

Recent work demonstrates that ssODNs can be used as a substitute for conventional plasmid-based targeting vectors as donor template for ZFN-assisted HR [26]. With the highly efficient design of ZFNc-kit, we tested whether ZFNc-kit could assist HR in mouse zygotes. Considering the fact that DSBs facilitate HR most efficiently near the nuclease cleavage site [28], we first attempted to insert a restriction site directly at the ZFNc-kit cleavage site in the mouse *c-kit* locus by ssODN. The ssODN donor template contained a 6 bp BglII site flanked by 25 bp of homology on each side (Figure 2A). 

The ssODNs donor template was co-injected with ZFNc-kit mRNAs into one-cell mouse embryos to generate transgenic mice. A total of 889 out of 1147 injected zygotes were transferred into 29 pseudopregnant females (Table 2). The resulting pups were genotyped by PCR-amplifying a 586 bp target region from tail genomic DNA obtained from 5-day-old pups and digested with BglII. The wild type *c-kit* allele contains no BglII restriction site, while the targeted mutant allele can be digested by BglII into two distinct fragments of 238 bp and 354 bp. As shown in Figure 2B, 10 out of 109 founders yielded BglII digested bands, indicating successful insertion of BglII restriction site. The PCR products of the 10 mutant founders were further cloned and sequenced to determine the precise location of the insertion. The sequencing results showed precise BglII insertions in 8 out of 10 mutant founders (Figure 2C), confirming successful targeted restriction site insertion by ZFNs and ssODN-mediated HR in mouse embryos.

**Table 2:** Summary of embryo microinjection of ZFN mRNA and ssODN.

ZFN mRNA	ssDNA	Embryos injected	Embryos transferred	Recipient	Pups	Pups with Bgl II or loxP site	Precise RH Rate	No. pups
5 ng/μl each	0.5 μM Bgl II-50H	260	208	8	13	1	0 (0/13)	#4-1 to #4-8, #5-1 to #5-5
5 ng/μl each	1 μM Bgl II-50H	341	257	7	16	2	6.25% (1/16)	#1-1 to #1-2, #2-1 to #2-3, #3-1 to #3-11
5 ng/μl each	0.5 μM Bgl II-100H	305	213	7	69	5	7.25% (5/69)	A1-A35, B1-B21, C1-C13
5 ng/μl each	1 μM Bgl II-100H	241	211	7	11	2	18.18% (2/11)	#6-1 to #6-4, #7-1 to #7-7
5 ng/μl each	1 μM loxP-80H	120	52	2	9	2	22.22% (2/9)	54-62

It is known that the use of longer homology arm results in a higher HR rate in conventional gene targeting [25]. To determine the effect of homology length and the concentration of ssODNs on targeting efficiency, we tested ssODNs donor with 25 or 50 bp homology length at concentration of 0.5 or 1.0 μM respectively (Table 2). The results showed that 50 bp homology at 1.0 μM yielded HR efficiency as high as 18.18%, whereas 25 bp homology at 0.5 μM did not induce any HR. Homology of 25 bp at 1.0 μM and 50 bp at 0.5 μM induced 6.25% and 7.25% HR, respectively. These results suggested that ZFNs and ssODN-mediated HR was ssODN homology length and concentration dependent.

### Targeted integration of a loxP into the c-kit locus by ZFNc-kit and gene-targeting ssODNs

**Figure 3:**
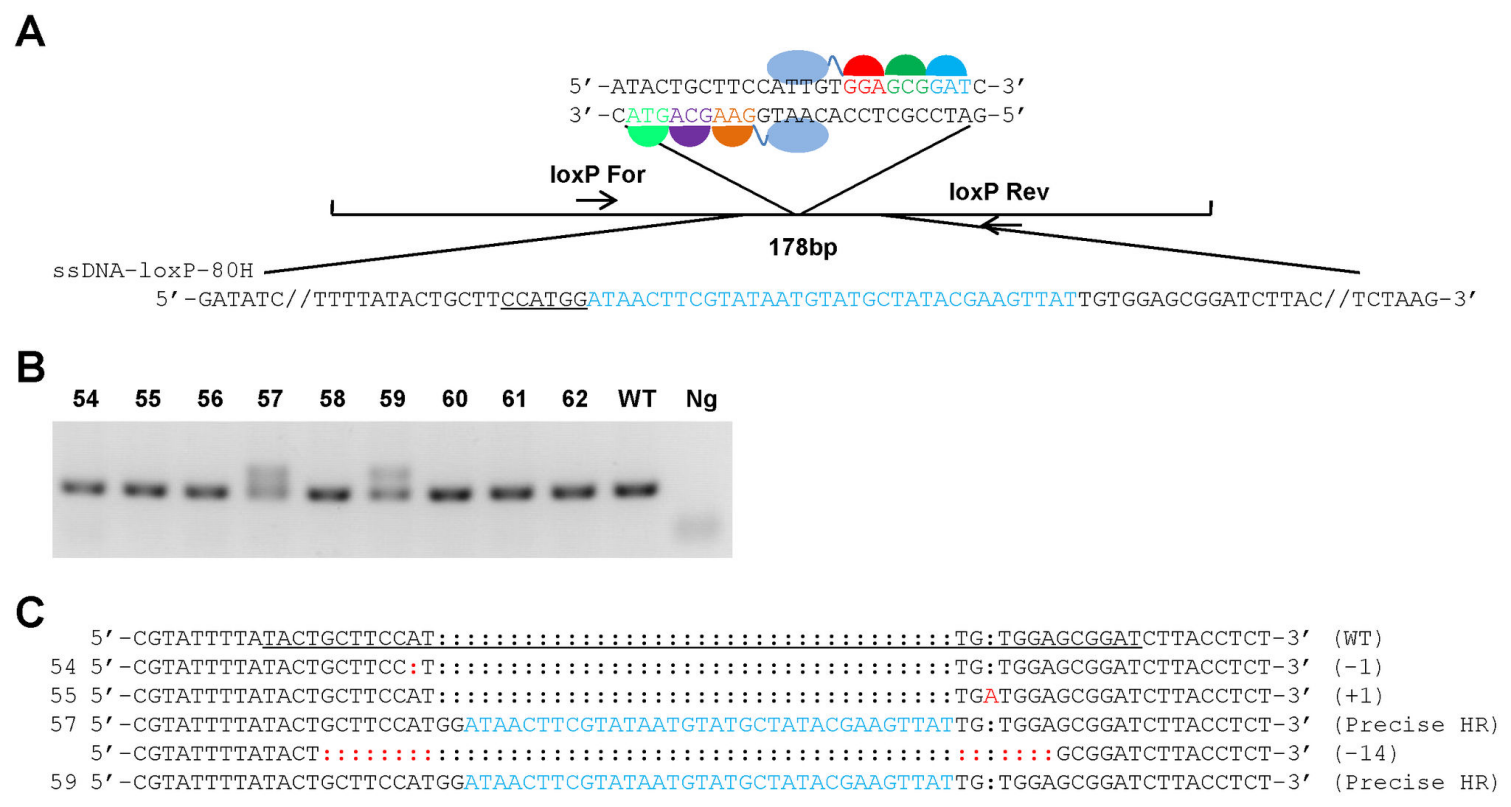
Targeted integration of a loxP site **using** **ZFNc-kit** and **gene-targeting** **ssODNs**. a. A schematic of the mouse *c-kit* locus and the ssODN sequences used to introduce a loxP site (in blue) into the genome *in* *vivo*. b. The representative gel electropherogram of the genotyping results by PCR amplification. 5 day old pups were genotyped by PCR amplification of tail genomic DNA. The expected wild type band is 178 bp (WT) and the expected mutant band is 214 bp. The PCR primers are listed in Table S1. Ng, negative control. c. Sequence analysis of the mutant *c-kit* allele of the 9 founders. The target region of the mouse *c-kit* gene was PCR-amplified from tail genomic DNA of the 5-day-old founders and subjected to T-A cloning. Colonies were randomly picked for DNA sequencing. Precise loxP site insertion was detected in 2 out of 9 founders. ZFN binding site is underlined. The loxP sites are highlighted in blue. Indels are highlighted in red. WT, wild type.

Encouraged by our success in targeted restriction site insertion by ZFNs and ssOND-mediated HR, we next explored the possibility of introducing a longer sequence such as a loxP site by ZFNs and ssODN-mediated HR. We tried to insert a loxP site directly into the ZFNc-kit cleavage site in the mouse *c-kit* locus using ssODN as donor template. The design of the ssODN contained a 34 bp loxP site flanked by 40 bp of homology on each side (Figure 3A). 

As described above, the ssODNs was co-injected with ZFNc-kit mRNAs into one-cell mouse embryos to generate transgenic mice. A total of 52 out of 120 injected zygotes were transferred into 2 pseudopregnant females (Table 2). The resulting 9 pups were genotyped by gel electrophoresis analysis of the PCR products of the target site from tail genomic DNA. The results indicate successful loxP site insertion in 2 out of 9 pups (Founder #57 & 59) (Figure 3B). Further characterization of the insertion site by sequencing confirmed precise loxP site integration in both founders 57 & 59 (Figure 3C), indicating an insertion efficiency of 22.22%. Moreover, we also observed indels in founders 54, 55, and 57 through analysis of T-A cloning products. These results demonstrate successful targeted loxP site insertion and the feasibility of precise genome editing by ZFNs and ssODN-mediated HR in mouse embryos.

### Knockin introduced by ZFNc-kit and gene-targeting ssODNs were heritable in mice

After the successful generation of mutant founder animals, we tested whether the targeting events could be transmitted through the germline to establish stable mouse lines. Three founders harboring the BglII restriction site (Founders C4, #7-1, A22) and 2 founders with the loxP insertion (Founders 57 & 59) were crossed to wild type mice for F1 mouse generation. 

**Figure 4:**
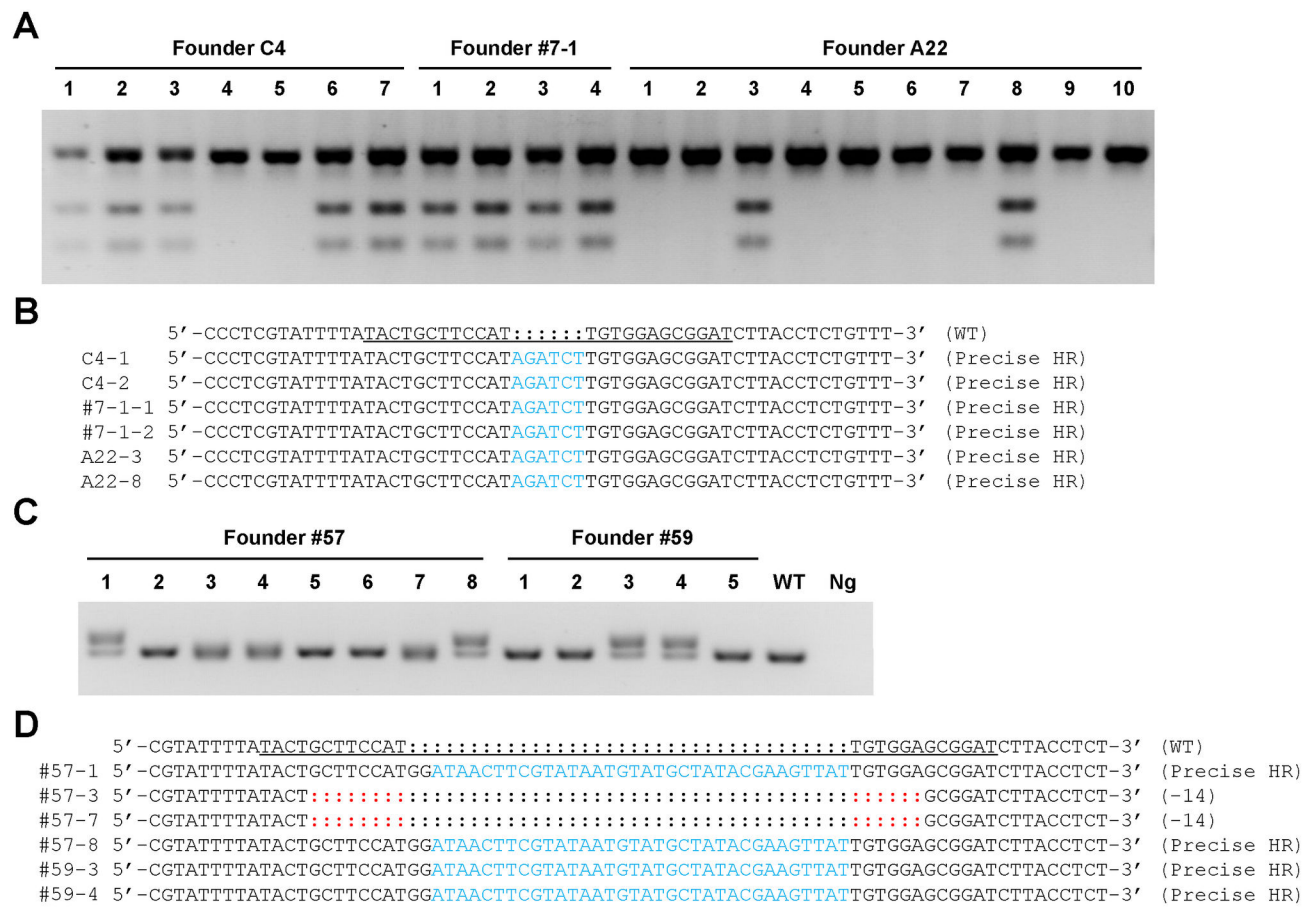
Germline transmission of the targeting modification. a. The representative gel electropherogram of the genotyping result by BglII digestion of the PCR product. F1 mice derived from founders C4, #7-1, and A22 were genotyped by BglII digestion of the PCR product (592 bp) amplified from tail genomic DNA of 5-day-old pups. The PCR product from the wild type *c-kit* allele cannot be digested by BglII (WT), while the targeted mutant allele can be digested by BglII into two distinct fragments (238 bp and 354 bp). The PCR primers are listed in Table S1. b. Sequence analysis of the BglII restriction site insertion. The F1 mice 1 & 2 from founder C4, #1 & 2 from founder 7-1, and #3 & 8 from founder A22 were selected for sequence analysis to confirm precise restrict site insertion. ZFN binding site is underlined. BglII sites are highlighted in blue. WT, wild type. c. The representative gel electropherogram of the genotyping results by PCR amplification. A total of 13 F1 mice from founder 57 & 59 with loxP integration were genotyped by PCR amplification using tail genomic DNA from 5-day-old pups. The expected wild type (WT) band is 178 bp, while the expected mutant band is 214 bp. The PCR primers are listed in Table S1. Ng, negative control. d. Sequence analysis of the mutant *c-kit* allele of the F1 mice 1, 3, 7, and 8 from founder 57, and #3 & 4 from founder 59. The targeted region of the mouse *c-kit* locus was PCR-amplified from tail genomic DNA of 5-day-old founders and subjected to sequencing. Precise loxP integration was detected in F1 mice 1 & 3 from founder 57, 3 & 4 from founder 59. ZFN binding site is underlined. loxP sites are highlighted in blue. Deletions are highlighted in red. WT, wild type.

A total 21 F1 mice from BglII insertion founders and 13 F1 mice from loxP integration founders (Figure 4A & C) were produced. The genotypes of the BglII insertion F1 offspring were determined by BglII digestion of PCR-amplified tail genomic DNA and sequencing as described above. Restriction analysis by BglII showed that 11 out of 21 F1 mice inherited the mutant allele (Figure 4A). F1 mice 1 & 2 from founder C4, #1 & 2 from founder 7-1, and #3 & 8 from founder A22 were selected for sequence analysis. As expected, sequencing confirmed inheritance of the precise BglII restrict site insertion in all F1 animals (Figure 4B). The genotypes of all the loxP integration F1 mice were determined by PCR. The mutant allele (214 bp) was detected in F1 mice 1 & 8 from founder 57 and 3 & 4 from founder 59 (Figure 4C). The precise loxP insertion was further confirmed by sequencing (Figure 4D). 

In short, both knockin mutant alleles were successfully and precisely transmitted to their offspring. This study demonstrates that genome editing by ZFN and ssODNs-assisted HR in mouse zygote is a robust strategy for producing germline competent mutant mouse models.

## Discussion

Nuclease-assisted genome editing in one-cell embryos can provide an easy route for creating knockout or knockin animal models in mice, rats, rabbits, and zebrafish [2-10,22-25,27]. The development of this technology has greatly facilitated the analysis of gene function in different animal species. Replacing conventional plasmid-based targeting vectors with ssODNs can further expedite the generation of mutant animal models, because ssODNs can be custom-synthesized quickly. Furthermore, the use of ssODNs reduces the potential for non-faithful integration compared with double-stranded oligodeoxynucleotides [26]. Although previous attempts with ZFN and ssODNs showed suboptimal performance in mouse [24], success in knockin by TALENs and ssODNs in zebrafish supports the possibility and necessity of further evaluation of the application of ZFNs and ssODNs [27]. Our study demonstrates seamless and efficient genome editing by ZFN and ssODNs-assisted HR in the mouse zygote by insertion of a restriction site and a loxP sequence in the mouse *c-kit* locus. 

As direct knockout of candidate genes can sometimes result in embryogenesis failure, conditional gene targeting using the Cre/loxP system is widely used to provide temporally regulated functional analysis *in vivo*. However, generation of conditional mutant allele by conventional Cre/loxP gene targeting is slow and expensive. Most importantly, it is currently only applicable in the mouse due to the lack of functional (germline competent) ES cell lines in other species [1]. The ZFN and ssODN-assisted HR strategy described in this report will not only save time and expense in generating loxP site mutant allele for Cre-dependent conditional genetic manipulation in mice, but will also provide a potential avenue for conditional genome editing in other animal models. We can envision that this strategy will facilitate precise engineering of mutant alleles for disease modeling. 

Of particular note, although it has been reported that ssODN assists nuclease-mediated HR in cultured cell lines at a similar rate to classical targeting vectors [26], our data suggest that ZFN and ssODN-assisted HR can be as high as 22.22% in mouse zygotes in comparison to 1.7~4.5% commonly seen in mouse oocytes using classical targeting vectors [5] and 2.7% in zebrafish oocytes by ssODNs [27]. The significant improvement of efficiency can be attributed to the design and optimization of our targeting strategy. We designed the site of insertion to coincide/overlap with the nuclease cleavage site. We also kept the length of both homology arms equal and isogenic to the endogenous target locus, instead of introducing silent mutations in the homology arms to protect the template donor from ZFN activity after successful HR [24,26,28]. We also optimized the concentration and length of the homology arms of the ssODNs donor. Interestingly, in contrast to the observation that longer homology arms decreases HR frequency in zebrafish [27], longer homology arms significantly improves HR in mouse, suggesting t the existence of differences in the HR machinery between species.

During the preparation of this manuscript, Dr. Kuhn’s group published their successes in generating a knockin mouse model at the efficiency of ~2% using TALEN and ssODNs [29], suggesting that TALEN combined with ssODN is also a feasible alternative for precise genome editing. In this study, our optimized design of the ssODN donor and ZFN cleavage site enabled us to achieve highly efficient nuclease-assisted HR up to 22.22%. Our study establishes a quick, simplified, and efficient genome editing strategy mediated by ZFN and ssODN-assisted HR in mouse zygotes.

## Methods

### Ethics Statement

Mice were housed in standard cages in an Assessment And Accreditation of Laboratory Animal Care credited SPF animal facility on a 12-h light/dark cycle. Wild type zebrafish used for laying were maintained at 28.5 °C in our zebrafish facility. Injected zebrafish embryos were cultured at 28.5 °C in an incubator. All animal protocols were approved by the Animal Care and Use Committee of the Model Animal Research Center, the host for the National Resource Center for Mutant Mice in China, Nanjing University.

### Design of ZFN Pair Targeting Mouse c-kit Gene

We utilized ZiFiT software which collects potential targeting sites in DNA sequences for ZFNs as our design tool (ZiFiT: http://zifit.partners.org/ZiFiT/) [30,31]. The design of ZFN pairs was assisted by SGMO module source with high GNN score and high affinity score [32], containing a 6 bp spacer flanked by two targeting sites and based on previous research results and our experience. DNA sequence of the 14^th^ intron of the mouse *c-kit* gene was the input used to search for potential targeting sites and their corresponding amino acid sequence for both left and right 3-zinc-finger arrays (Figure S1A). The designed ZFN pairs were synthesized and then cloned into plasmids (left arm in pST1374-c-kit-L; right arm in pCDNA3.1-c-kit-R) [33,34] under T7 promoter by Taihe Gene (Beijing). The ZFN amino acid sequences are listed in Sequences S1.

### Preparation of ZFN targeting vector and ZFNc-kit mRNA

The activity of ZFNc-kit was first tested t in zebrafish embryos to examine its targeting efficiency on a naked plasmid targeting site. We cloned the genomic DNA sequence of the mouse *c-kit* gene targeting site into pMD18-T Simple Vector (TaKaRa, D103A) with a pair of primers positioned outside of the corresponding target sequence: CK14 check ZFN For and Rev (Table S1). The resulting plasmid (pMD18-T-mouse c-kit) was extracted by phenol and chloroform to render it RNase free. pST1374-c-kit-L was digested by AgeI and pCDNA3.1-c-kit-R was digested by EcoRI to linearize the plasmids and *in vitro* transcribed to generate ZFNc-kit mRNA (capped and tailed) using mMessage mMachine T7 Ultra Kit (Ambion, AM1345). The mRNA was purified using an RNeasy Mini Kit (Qiagen, 74104). The mRNA concentration and quality were determined by absorption and gel analysis. The ZFNc-kit mRNA was divided into aliquots and stored at -80 °C. Primers used are listed in Table S1.

### Test of ZFNc-kit activity in zebrafish

The mRNAs of the left and right arm of ZFN were mixed with plasmid pMD18-T-mouse c-kit to a final concentration of 200 ng/μl mRNA per ZFN arm and 25 ng/μl pMD18-T-mouse c-kit. 0.5 nl of the mixture was microinjected into zebrafish embryos at the 1-2 cell stage. The injected embryos were cultured at 28.5 °C and collected 24 hours post fertilization for DNA extraction. The extracted DNA was dissolved in 200 μl water and 1 μl of the solution was used as template to amplify the ZFNc-kit targeting site by PCR with the same primers used for target site cloning described above. Twenty cycles of amplification were done to preserve the mutations by nonhomologous end joining repair caused by ZFNc-kit and minimize amplification error due to PCR. The PCR product was then cloned into the pMD18-T Simple Vector and transformed into DH5α competent cells. Colonies were randomly picked using blue-white screening and 80 positive colonies were sequenced to verify nuclease activity of ZFNc-kit. The sequences were then aligned with the wild type mouse *c-kit* sequence to determine specific mutations.

### Preparation of ssODN

The ssODNs donor templates (Sequences S1) were synthesized as normal oligonucleotides and purified by PAGE (Life Technologies). ssODNs were diluted with RNase free water to 100 μM, divided into aliquots and stored at -20 °C. 

### ZFNc-kit mRNA and ssODN injection of one-cell embryos

Mouse zygotes were obtained by mating of CBA males with superovulated C57BL/6J females as described [5]. Briefly, zygotes were injected with ZFNc-kit mRNA (2.5 or 5 ng/μl) or a mixture of the donor ssODNs (0.5 or 1 μM) and ZFNc-kit mRNA (5 ng∕μl). Microinjections were performed into the larger (male) pronucleus of fertilized oocytes and cytoplasm. Injected zygotes were transferred into pseudopregnant CD1 female mice, and viable adult mice were obtained. Founder animals with the correctly targeted mutant allele were mated, and their offspring were genotyped for germline transmission. All mice appeared healthy and showed normal development. 

### T7EN1 cleavage assay

T7EN1 cleavage assay was performed as described [35]. In brief, targeted fragments were amplified from extracted DNA, and purified using a PCR cleanup kit (Axygen, AP-PCR-50). Purified PCR products were denatured and re-annealed in NEB buffer 2 (NEB) using a thermocycler. Hybridized PCR products were digested with T7EN1 (NEB, M0302L) for 30 min and analyzed on a 2% agarose gel.

### PCR and sequence analysis

Injected zebrafish embryos cultured for 12 hours or tail tips from 5 day-old-mice were incubated in 500 μl lysis buffer (10 M Tris-HCl, 0.4 M NaCl, 2M EDTA, 1% SDS and 100 g/ml Proteinase K) at 55 °C overnight. DNA was purified from lysates by phenol-chloroform extraction. The DNA pellets were dissolved in water, and stored at 4 °C. The extracted DNA was used as template for subsequent PCR amplification. PCR products or T-A colonies cloned from PCR products were sent for sequencing to identify the presence of indels or mutations. The resulting sequences were aligned with corresponding wild type sequence using the Vector NTI software (Invitrogen).

## Supporting Information

Figure S1 (TIFF)

Table S1 **Primers for genotyping and amplifying ZFN target fragments.**
(DOC)

Sequences S1 Supporting sequences.
(DOC)
