# Supplementary figures and images for: Efficient Knockin Mouse Generation by ssDNA Oligonucleotides and Zinc-Finger Nuclease Assisted Homologous Recombination in Zygotes
Source: PLoS One. 2013 Oct 22;8(10):e77696. doi: 10.1371/journal.pone.0077696 (PMC3805579; doi:10.1371/journal.pone.0077696)

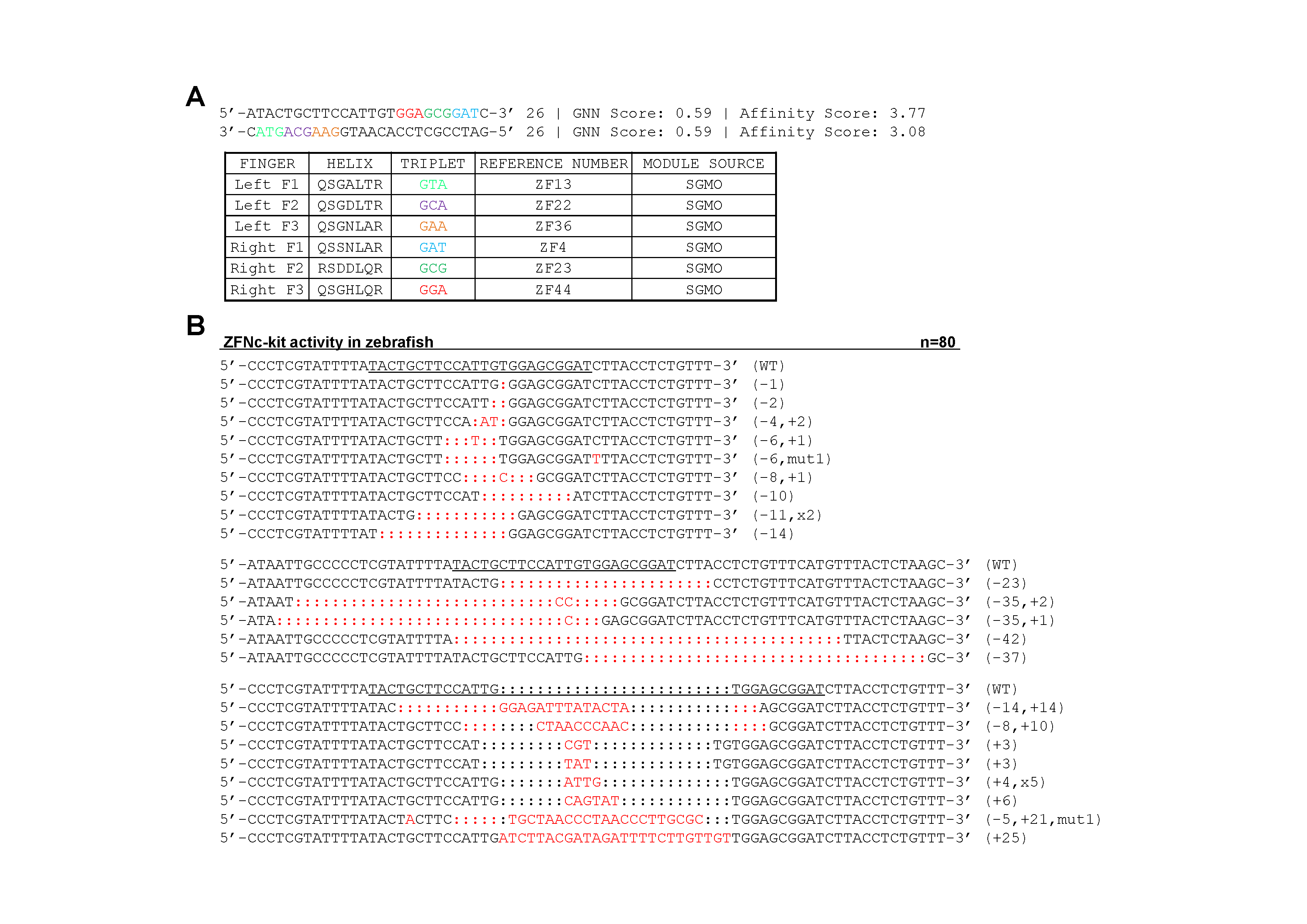

Supplement: Figure S1 — (TIFF) [file pone.0077696.s001.tiff]
