# Supplementary material for: Efficient Knockin Mouse Generation by ssDNA Oligonucleotides and Zinc-Finger Nuclease Assisted Homologous Recombination in Zygotes
Source: PLoS One. 2013 Oct 22;8(10):e77696. doi: 10.1371/journal.pone.0077696 (PMC3805579; doi:10.1371/journal.pone.0077696)
Supplement: Table S1 — Primers for genotyping and amplifying ZFN target fragments. (DOC) [file pone.0077696.s002.doc]

| Primers | Sequence | Amplicon |
| --- | --- | --- |
| CK14 check ZFN For | accacccagcatatgtagactac | 586 bp |
| CK14 check ZFN Rev | AAGCATGAGGCTTCTCTTGGGAC |
| ck14 check HR Loxp For | ctttcttccccaatgtgcttgag | WT, 178 bp Mut, 214 bp |
| ck14 check HR Loxp Rev | GACAGTTGGTATGGCATTTCATG |

**Table S1** | Primers for genotyping and amplifying ZFN target fragments
