# Supplementary material for: Efficient Knockin Mouse Generation by ssDNA Oligonucleotides and Zinc-Finger Nuclease Assisted Homologous Recombination in Zygotes
Source: PLoS One. 2013 Oct 22;8(10):e77696. doi: 10.1371/journal.pone.0077696 (PMC3805579; doi:10.1371/journal.pone.0077696)
Supplement: Sequences S1 — Supporting sequences. (DOC) [file pone.0077696.s003.doc]

**Supplementary Sequences**

**ZFNc-Kit left arm** (310aa)

MGPKKKRKVAAADYKDDDDKSRLEPGEKPYKCPECGKSFSQSGALTRHQRTHTGEKPYKCPECGKSFSQSGDLTRHQRTHTGEKPYKCPECGKSFSQSGNLARHQRTHTGKKTSQLVKSELEEKKSELRHKLKYVPHEYIELIEIARNSTQDRILEMKVMEFFMKVYGYRGKHLGGSRKPDGAIYTVGSPIDYGVIVDTKAYSGGYNLPIGQADEMQRYVKENQTRNKHINPNEWWKVYPSSVTEFKFLFVSGHFKGNYKAQLTRLNHKTNCNGAVLSVEELLIGGEMIKAGTLTLEEVRRKFNNGEINF

**ZFNc-Kit right arm** (310aa)

MGPKKKRKVAAADYKDDDDKSRLEPGEKPYKCPECGKSFSQSSNLARHQRTHTGEKPYKCPECGKSFSRSDDLQRHQRTHTGEKPYKCPECGKSFSQSGHLQRHQRTHTGKKTSQLVKSELEEKKSELRHKLKYVPHEYIELIEIARNSTQDRILEMKVMEFFMKVYGYRGKHLGGSRKPDGAIYTVGSPIDYGVIVDTKAYSGGYNLPIGQADEMERYVEENQTRNKHLNPNEWWKVYPSSVTEFKFLFVSGHFKGNYKAQLTRLNHITNCNGAVLSVEELLIGGEMIKAGTLTLEEVRRKFNNGEINF

**ssDNA-BglII-50H**

ccctcgtattttatactgcttccatAGATCTtgtggagcggatcttacctctgttt

**ssDNA-BglII-100H**

aggcccagtggatatcataattgccccctcgtattttatactgcttccatAGATCTtgtggagcggatcttacctctgtttcatgtttactctaagccccgggttc

**ssDNA-loxP-80H**

gatatcataattgccccctcgtattttatactgcttccatggATAACTTCGTATAATGTATGCTATACGAAGTTATtgtggagcggatcttacctctgtttcatgtttactctaag
